# Scanning Electron Microscope Study of Antennae and Mouthparts in the Pollen-Beetle *Meligethes* (*Odonthogethes*) *chinensis* (Coleoptera: Nitidulidae: Meligethinae)

**DOI:** 10.3390/insects12070659

**Published:** 2021-07-20

**Authors:** Qihang Li, Longyan Chen, Meike Liu, Wenkai Wang, Simone Sabatelli, Andrea Di Giulio, Paolo Audisio

**Affiliations:** 1 Institute of Entomology, College of Agriculture, Yangtze University, Jingzhou 434025, China; l13177033833@163.com (Q.L.); clylyly@126.com (L.C.); w_wenkai@hotmail.com (W.W.); 2 Department of Biology and Biotechnology, Sapienza Rome University, Viale dell’Università 32, 00185 Rome, Italy; paolo.audisio@uniroma1.it; 3 Department of Science—L.I.M.E., University of Roma Tre, V.le G. Marconi, 446, 00146 Rome, Italy; andrea.digiulio@uniroma3.it

**Keywords:** Cucujoidea, phytophagy, sexual dimorphism, fine morphology

## Abstract

**Simple Summary:**

The present paper is aimed to further explore the structure of the antennae and mouthparts of specialized beetle species living on flowers, as well as the functions of their associated sensilla. In this study, we used scanning electron microscopy (SEM) to observe and describe for the first time the fine morphology of sensilla on the antennae and mouthparts of the pollen-beetle *Meligethes* (*Odonthogethes*) *chinensis*, a common Chinese species associated with flowers of Rosaceae. The results show that there are six types and twelve subtypes of sensilla on male antennae; seven types and fourteen subtypes on female antennae; seven types and seventeen subtypes on male mouthparts; seven types and sixteen subtypes on female mouthparts. The sensilla on the antennae and mouthparts of Meligethinae that feed on pollen were finally compared with similar sensilla known to occur in other insects, in order to obtain more insights on the evolution of these sensorial structures in specialized flower-inhabiting insects.

**Abstract:**

*Meligethes* (*Odonthogethes*) *chinensis* is a common Chinese phytophagous species in the family Nitidulidae. Its main larval host plant is *Rubus idaeus* L. (Rosaceae), and adults feed on pollen and other floral parts. In this study, we used scanning electron microscopy (SEM) to observe and study the fine morphology of sensilla on the antennae and mouthparts of *M. chinensis*. The results show that there are six types and twelve subtypes of sensilla on male antennae; seven types and fourteen subtypes on female antennae; seven types and seventeen subtypes on male mouthparts; seven types and sixteen subtypes on female mouthparts. Sensilla coeloconica (SCo) are found on the female antennae of *M. chinensis* only, and they are also reported on the antennae of Nitidulidae for the first time. SCo2 on the labrum present sexual dimorphism, and one subtype of sensilla basiconica (SB6) is presented on the tip of maxillary and labial palps of the male only, while other types of sensilla are very similar on the mouthparts of male and female. Finally, by comparing similar sensilla in other insects, we also attempted to discuss the functions of all sensilla on the antennae and mouthparts of *M. chinensis*.

## 1. Introduction

Nitidulidae is a family of the diverse superfamily Cucujoidea, including about 4500 species in the world, with heterogeneous feeding habits, such as phytophagy, saprophagy, and micetophagy [1,2,3,4]. Meligethinae is the only subfamily among Nitidulidae strictly anthophagous, with members commonly known as “pollen-beetles”; in fact, all members (about 700 species all over the world) of this lineage use pollen and other floral parts as larval and adult food resource [5,6,7,8,9,10,11]. Anthophagy independently evolved in members of the related Neotropical tribe Mystropini in the subfamily Nitidulinae [1]. *Meligethes* (*Odonthogethes*) *chinensis* is a common Chinese phytophagous species in the subfamily Meligethinae. Its main larval host plant is *Rubus idaeus* L. (Rosaceae), and adults mainly feed on the pollen of this plant [7,11]. This species exhibits an apparently marked variation in body size, shape, pubescence, and color, and females are usually longer than males [9].

The sensilla on antennae and mouthparts are important structures for both consumption and location of their specialized food resources. Due to the limited number of comparative studies, the sensory diversity of these structures involving communication and feeding behavior is still poorly understood, and such studies often focus on only one sex or do not emphasize differences between the two sexes [12]. While, in addition to finding and identifying food resources, mating partners, and finding suitable habitats like males, females of pollen-beetles Meligethinae also need to find a suitable oviposition site on the host plants [1,2,13]. For these reasons, it is necessary to study in detail the types, quantities, and distributions of the sensilla of pollen-beetles of both sexes. However, among Nitidulidae at present, the head sensilla have been investigated with modern criteria only in the zoosaprophagous *Omosita colon* (Nitidulinae) [14], while antennal sensilla of other Nitidulidae species exhibiting a different lifestyle and diet have been only poorly studied thus far [1,15].

In this study, the pollen-beetle *M. chinensis* was selected to study types, quantities, and distributions of sensilla on the antennae and mouthparts of males and females, respectively, for the first time by scanning electron microscopy (SEM), and to be compared with the sensilla described in the zoosaprophagous *O. colon* [14]. We present this comparative study on male and female *M. chinensis*, thus, not only having the first Nitidulidae lineage with both sexes analyzed in detail but also helping to understand the evolution of sensillar equipment, because if the stimuli that guide the male and female activities are different, then it is completely predictable that there will be present sex dimorphism in the sensillar equipment [16]. Finally, by comparing similar sensilla in other insects, we also tried to initially discuss the functions of all sensilla on the antennae and mouthparts of *M. chinensis*. This paper will provide a basis for further studies on the relationship between sensilla and behavior of this insect.

## 2. Materials and Methods

### 2.1. Specimens

Adults of *Meligethes* (*Odonthogethes*) *chinensis* for this study were collected on flowers of *Rubus idaeus* L. (Rosaceae) by the corresponding author ML in the Shennongjia Forestry District of Hubei province in July, 2020. The specimens were preserved in EP tubes with 70% ethanol and stored at −20 °C for later use.

### 2.2. Comparative Morphological Research

#### 2.2.1. Specimens Selection, Dissections, and Imaging

In this study, we took eight male and eight female adults of *M. chinensis*. Antennae and mouthparts were dissected from each specimen by using tweezers and needles. Labrum, mandibles, maxillae, and labium were detached from each specimen under a stereomicroscope.

#### 2.2.2. Fine Morphological Research

The antennae and the mouthparts of the male and female adults were preserved in 70% ethanol. We used the ultrasonic cleaning machine to clean the samples for 10 min twice. Then, they were dehydrated for 20 min in 70%, 80%, 85%, 90%, and 95% ethanol gradients, respectively, and immersed in 100% ethanol for 30 min twice, then treated with pure tert-butanol for 30 min. The specimens were placed into a Christ-Alpha 1-2 LD freeze-drier for 2 h. After drying, they were fixed on the sample table with conductive glue and sputtered with gold using a Quorum-SC7620 sputter instrument. Finally, all specimens of *M*. *chinensis* were observed and photographed under a Tescan-Vega 3 SBU scanning electron microscope operated at 20 kV.

### 2.3. Data Analysis

The naming of sensilla types was based on the classification system method of Schneider [17]. According to the SEM micrographs, the length of each antennal segment and the length, quantity, and distribution of each type of sensilla from eight male and eight female adult specimens of *M*. *chinensis* were obtained by using Image J [18]. The length and base diameter of each type of sensilla were measured from at least eight individuals and processed as mean ± SE. The data about the total antenna length of male and female adults were tested by t-test with SPSS 17.0 (http://www.spss.com, accessed on 10 June 2021) to determine whether the difference was significant in experimental data (*p* < 0.05).

## 3. Results

### 3.1. Typology, Characteristics, and Distribution of the Antennal Sensilla

**Figure 1:**
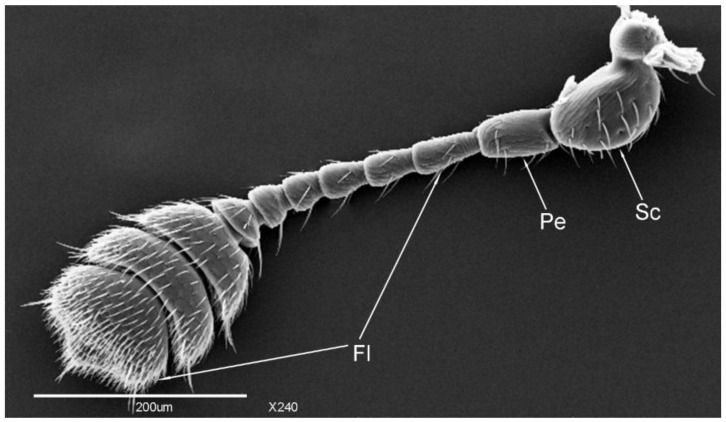
The whole view of the antenna of female *Meligethes* (*Odonthogethes*) *chinensis*. Sc, scape; Pe, pedicel; Fl, flagellum.

The length of total antenna of female *Meligethes* (*Odonthogethes*) *chinensis* was significantly longer than that of male (*n* = 8, *t* = 6.148, *p* < 0.05). In general, the females were frequently slightly larger (on the average) than males in *Meligethes*. In this study, the body lengths of the female and male adults were, respectively, 3.1 ± 0.13 mm and 2.8 ± 0.18 mm. Therefore, it is normal that the antennae of females were also proportionally longer. The antennal lengths were 593.96 ± 9.65 μm in females and 565.37 ± 3.76 μm in males (Table 1). The antenna has 11 segments: scape, pedicel, and nine-segmented flagellomeres (Figure 1). The antennae are rodlike, and the three terminal segments combined together are called the antennal club. The quantity and types of sensilla on the dorsal view of the antennae are greater than on the ventral view, whereas the quantity of dorsal and ventral sensilla is largest in the three terminal segments of the flagellomeres (antennal club). There are many types of sensilla in the scape and antennal terminal segment, and the sensilla are intensively presented on the antennal terminal segment.

**Figure 2:**
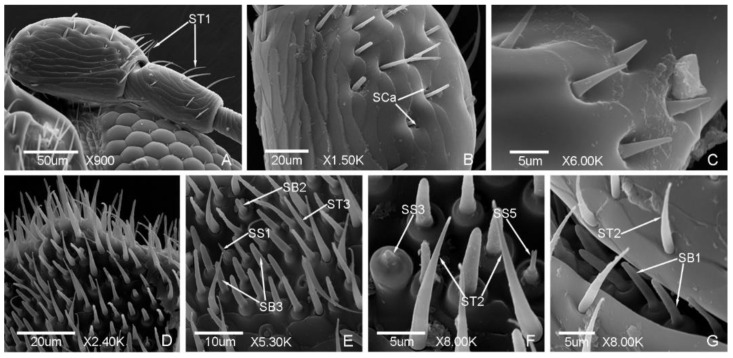
Antennal sensilla in the male of *Meligethes* (*Odonthogethes*) *chinensis*. (**A**) The dorsal view of the scape and pedicel. ST1, sensilla trichodea 1; (**B**) SCa, sensilla cavity on the scape; (**C**) BB, Böhm bristles on the pedicel; (**D**) antennal terminal segment; (**E**,**F**) the sensilla on the antennal terminal segment. ST2, ST3, sensilla trichodea 2, 3; SB2, SB3, sensilla basiconica 2, 3; SS1, SS3 and SS5, sensilla styloconica 1, 3 and 5; (**G**) SB1, sensilla basiconica 1 on the internode of the antennal club.

**Figure 3:**
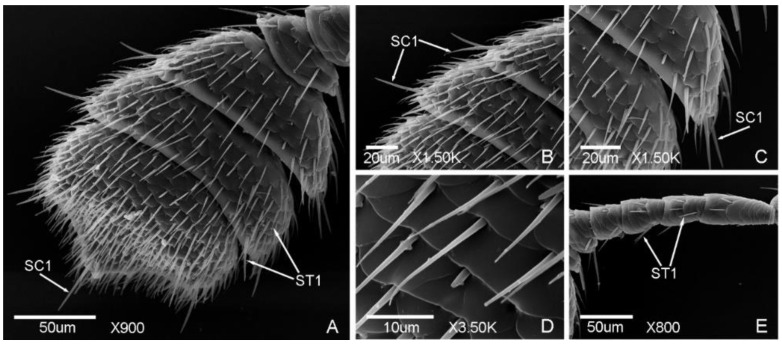
Antennal sensilla in the male of *Meligethes* (*Odonthogethes*) *chinensis*. (**A**) Antennal club. ST1, sensilla trichodea 1; SC1, sensilla chaetica 1; (**B**–**D**) SC1 and ST1 on the antennal club; (**E**) ST1 on flagellomeres.

**Figure 4:**
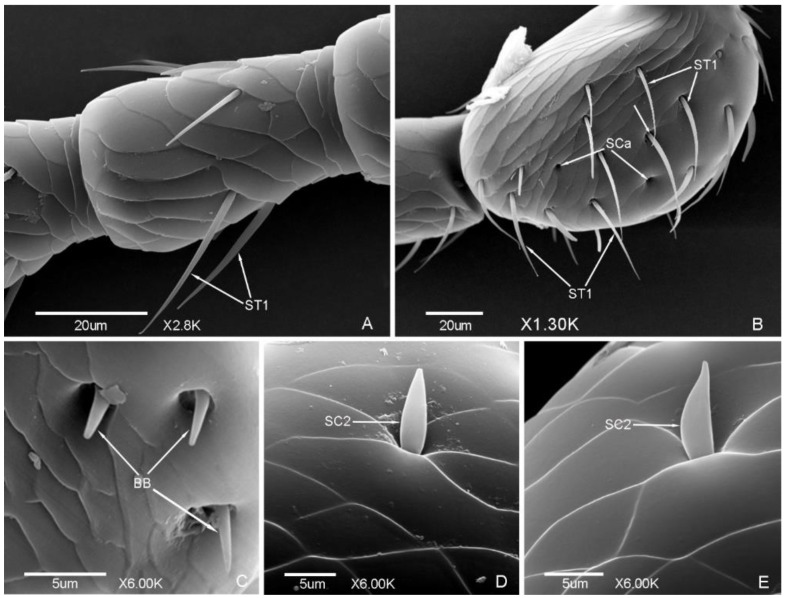
Antennal sensilla in the female of *Meligethes* (*Odonthogethes*) *chinensis*. (**A**) ST1, sensilla trichodea 1 on the flagellum; (**B**–**E**) the sensilla on the scape. ST1; SCa, sensilla cavity; BB, Böhm bristles; SC2, sensilla chaetica 2.

**Figure 5:**
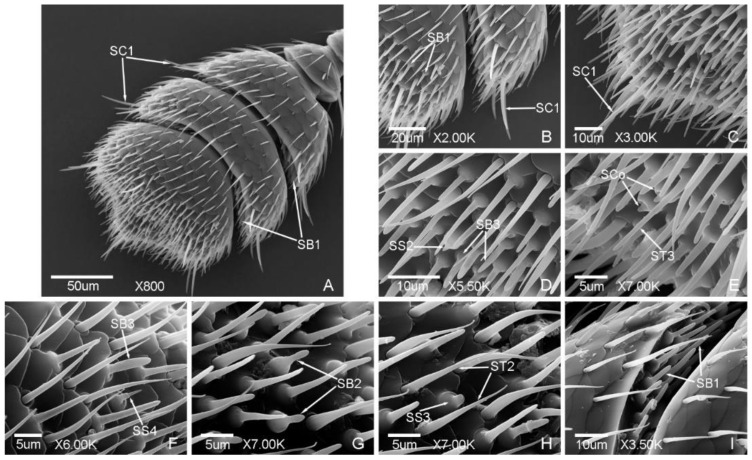
Antennal sensilla in the female of *Meligethes* (*Odonthogethes*) *chinensis*. (**A**) Antennal club. SC1, sensilla chaetica 1; SB1, sensilla basiconica 1; (**B**) the sensilla on the antennal club. SC1; SB1; (**C**–**H**) the sensilla on the antennal terminal segment. SC1; SS2, SS3, SS4, sensilla styloconica 2, 3, 4; SB3, sensilla basiconica 3; ST3, sensilla trichodea 3; SCo, Sensilla coeloconica; SB2, sensilla basiconica 2; ST2, sensilla trichodea 2; (**I**) SB1 on the internode of the antennal club.

Seven types of sensilla presented on the antennae of the male (Figure 2 and Figure 3) and female adults (Figure 4 and Figure 5), composed of sixteen subtypes, including sensilla trichodea (three subtypes), sensilla chaetica (two subtypes), sensilla basiconica (three subtypes), Böhm bristles, sensilla styloconica (five subtypes), sensilla cavity, and sensilla coeloconica (Table 2; Figure 2, Figure 3, Figure 4 and Figure 5). There are obvious differences in the types of antennal sensilla between the males and females, the types of antennal sensilla in females being more numerous than in males. Among the sensilla, sensilla coeloconica (SCo), sensilla chaetica (SC2), and sensilla styloconica (SS2, SS4) were found in the females only; sensilla styloconica (SS1, SS5) were found in the males only.

#### 3.1.1. Sensilla Trichodea (ST)

ST are long and thin, nearly straight or slightly curved, presented in large quantity, and found in both males and females. Three subtypes were found in this study.

ST1 are thin, slightly curved, with a slightly grooved surface, a sharp and thin tip, and shallow socket. ST1 are presented on each segment of the antennae (Figure 2a, Figure 3d,e and Figure 4a,b). The length was 24.35 ± 3.30 μm and the base diameter was 1.30 ± 0.12 μm.

ST2 are thicker than ST1, curved in arc shape, with a distinct grooved surface. ST2 are mainly presented on the antennal club (Figure 2f,g and Figure 5h). The length was 13.41 ± 1.80 μm and the base diameter was 1.67 ± 0.11 μm.

ST3 are thin and straight, with a smooth surface, blunt tip, and shallow socket. ST3 are mainly presented on the antennal terminal segment (Figure 2e and Figure 5e). The length was 14.91 ± 1.09 μm and the base diameter was 2.12 ± 0.13 μm.

#### 3.1.2. Sensilla Chaetica (SC)

SC are straight, with a sharp tip and shallow socket. According to the length of the sensilla, SC were divided into two subtypes. SC1 were found in both males and females, while SC2 were found in females only.

SC1 are obviously longer than other types of sensilla, bristly, with a grooved surface and tapering tip. SC1 are presented on the lateral side of the antennal club of the males and females (Figure 3b,c and Figure 5b,c). The length was 25.82 ± 0.83 μm and the base diameter was 2.50 ± 0.10 μm.

SC2 are short, leaf-like, with a smooth surface and slightly sharp tip. SC2 are presented on the lateral side of the scape of the females only (Figure 4d,e). The length was 7.66 ± 0.28 μm and the base diameter was 2.11 ± 0.16 μm.

#### 3.1.3. Sensilla Basiconica (SB)

SB are thick, straight or slightly curved, with a blunt tip. SB are located on the round base and the shallow socket (Figure 2e,g). Three subtypes were found in this study.

SB1 are straight or slightly curved, with a smooth surface and blunt tip. SB1 are mainly presented on the internodes of the antennal club (Figure 2g and Figure 5i) and on the surface of the antennal terminal segment (Figure 5b). The length was 7.66 ± 0.65 μm and the base diameter was 2.94 ± 0.17 μm.

SB2 are straight, with a shallow surface and blunt tip. The middle part is thinner than the terminal. SB2 are presented on the antennal terminal segment (Figure 2e and Figure 5g). The length was 5.62 ± 0.38 μm and the base diameter was 2.93 ± 0.11 μm.

SB3 are straight, with a slightly grooved surface and blunt tip. SB3 are presented on the antennal terminal segment (Figure 2e and Figure 5d,f). The length was 7.03 ± 0.53 μm and the base diameter was 2.99 ± 0.23 μm.

#### 3.1.4. Böhm Bristles (BB)

BB are short and straight, with no hole, a smooth surface, sharp tip, and deep socket. BB are presented on the scape and pedicel of both males and females (Figure 2c and Figure 4c). The length was 4.84 ± 0.65 μm and the base diameter was 1.47 ± 0.11 μm.

#### 3.1.5. Sensilla Styloconica (SS)

SS are a cone-like peg with a smooth surface, and located on the round base and shallow socket (Figure 2f). According to the length and the tip shape of the sensilla, they can be divided into five subtypes. SS1, SS3, and SS5 were found on the male antennae, while SS2, SS3, and SS4 were found on the female antennae.

SS1 are long and thick, with a conical tip, and presented on the antennal terminal segment of the males (Figure 2e). The length was 4.22 ± 0.21 μm and the base diameter was 2.45 ± 0.03 μm.

SS2 are slightly shorter than SS1, with a small protrusion on the tip, and presented on the antennal terminal segment of the females (Figure 5d). The length was 2.64 ± 0.11 μm and the base diameter was 2.56 ± 0.09 μm.

SS3 are short and thick, with a blunt tip, and presented on the antennal terminal segment of both males and females (Figure 2f and Figure 5h). The length was 1.26 ± 0.16 μm and the base diameter was 3.38 ± 0.28 μm.

SS4 are long, straight, grooved, and nailed. There is an obvious segmentation at halfway, and the tip is long and pointed. SS4 are presented on the antennal terminal segment of the females (Figure 5f). The length was 4.18 ± 0.05 μm and the base diameter was 3.03 ± 0.16 μm.

SS5 are similar with SS4 but with a bifurcated tip, and presented on the antennal terminal segment of the males (Figure 2f). The length was 3.10 ± 0.05 μm and the base diameter was 2.92 ± 0.10 μm.

#### 3.1.6. Sensilla Cavity (SCa)

The shape of SCa is a concave cavity, and the quantity is small. SCa are presented on the lateral side of the scape of both males and females (Figure 2b and Figure 4b). The diameter was 2.03 ± 0.12 μm.

#### 3.1.7. Sensilla Coeloconica (SCo)

SCo are short, straight, and wide, with a smooth surface and conical blunt tip, and the base is attached on the circular socket. SCo are presented on the antennal terminal segment of the females only (Figure 5e). The length was 1.53 ± 0.03 μm and the base diameter was 2.81 ± 0.15 μm.

### 3.2. Typology, Characteristics and Distribution of Sensilla on the Mouthparts

**Figure 6:**
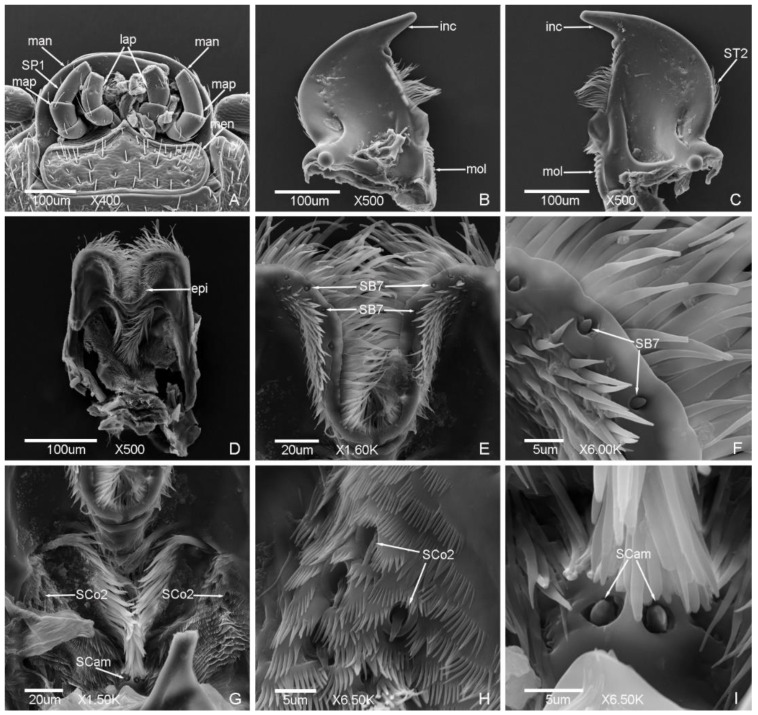
Sensilla on the labrum and mandible of *Meligethes* (*Odonthogethes*) *chinensis* male. (**A**) The ventral view of mouthpart. man, mandible; map, maxillary palps; lap, labial palps; men, mentum; SP1, sensilla placodea 1; (**B**,**C**) the ventral view of mandible. inc, incisor lobe; mol, molar lobe; ST2, sensilla trichodea 2; (**D**) the ventral view of labrum. epi, epipharynx; (**E**,**F**) SB7, sensilla basiconica 7 on the epipharynx; (**G**–**I**) the sensilla on the labrum. SCo2, sensilla coeloconica 2; SCam, sensilla campaniformia.

**Figure 7:**
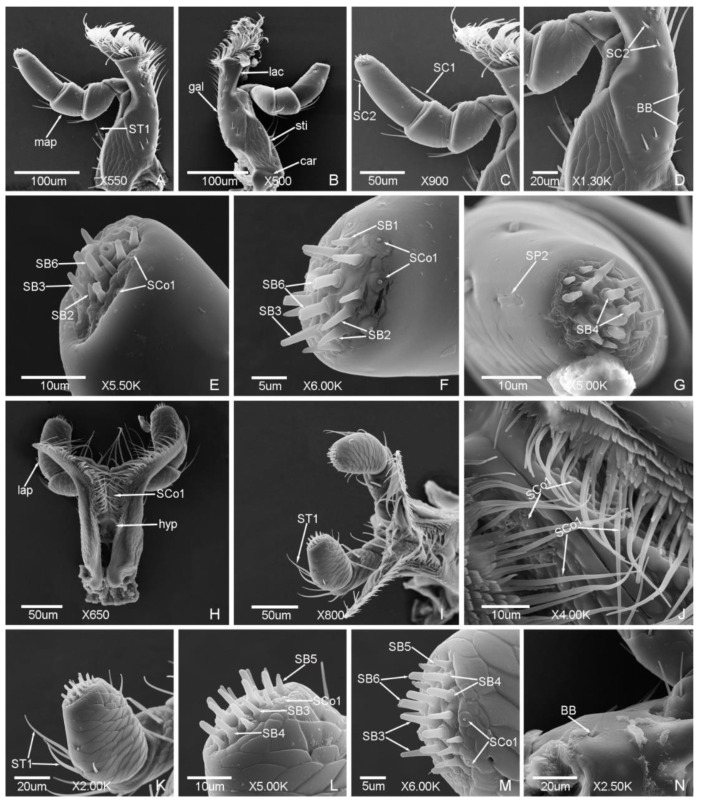
Sensilla on the maxillae and labium of *Meligethes* (*Odonthogethes*) *chinensis* male. (**A**,**B**) The ventral view of maxillae. map, maxillary palps; car, cardo; sti, stipes; gal, galea; lac, lacinia; ST1, sensilla trichodea 1; (**C**) the sensilla on the maxillary palps. SC1, SC2, sensilla chaetica 1, 2; (**D**) the sensilla on the galea. SC2; BB, Böhm bristles; (**E**–**G**) the sensilla on the tip of the terminal segment of maxillary palps. SB1, SB2, SB3, SB4 and SB6, sensilla basiconica 1, 2, 3, 4 and 6; SCo1, sensilla coeloconica 1; SP2, sensilla placodea 2; (**H**–**K**) the dorsal view of labium. lap, labial palps; hyp, hypopharynx; SCo1; ST1; (**L**,**M**) the sensilla on the tip of the terminal segment of labial palps. SCo1; SB3, SB4, SB5 and SB6, sensilla basiconica 3, 4, 5 and 6; (**N**) BB on the labium.

**Figure 8:**
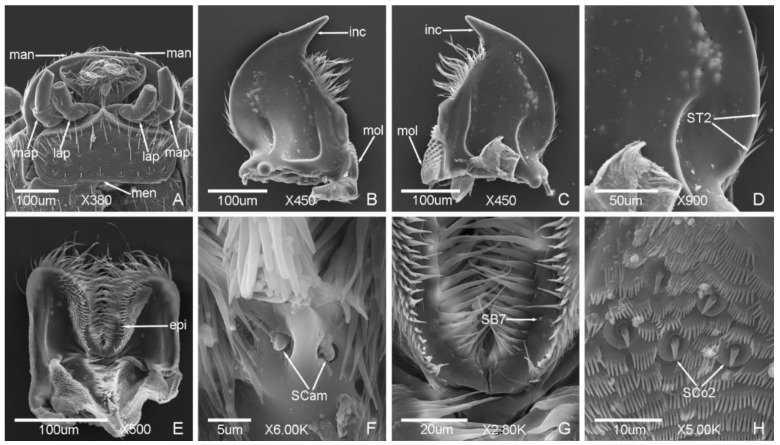
Sensilla on the labrum and mandible of *Meligethes* (*Odonthogethes*) *chinensis* female. (**A**) Ventral view of mouthpart. man, mandible; map, maxillary palps; lap, labial palps; men, mentum; (**B**–**D**) the ventral view of mandible. inc, incisor lobe; mol, molar lobe; ST2, sensilla trichodea 2; (**E**) the ventral view of labrum. epi, epipharynx; (**F**) SCam, sensilla campaniformia on the labrum; (**G**) SB7, sensilla basiconica 7 on the epipharynx; (**H**) SCo2, sensilla coeloconica 2 on the labrum.

**Figure 9:**
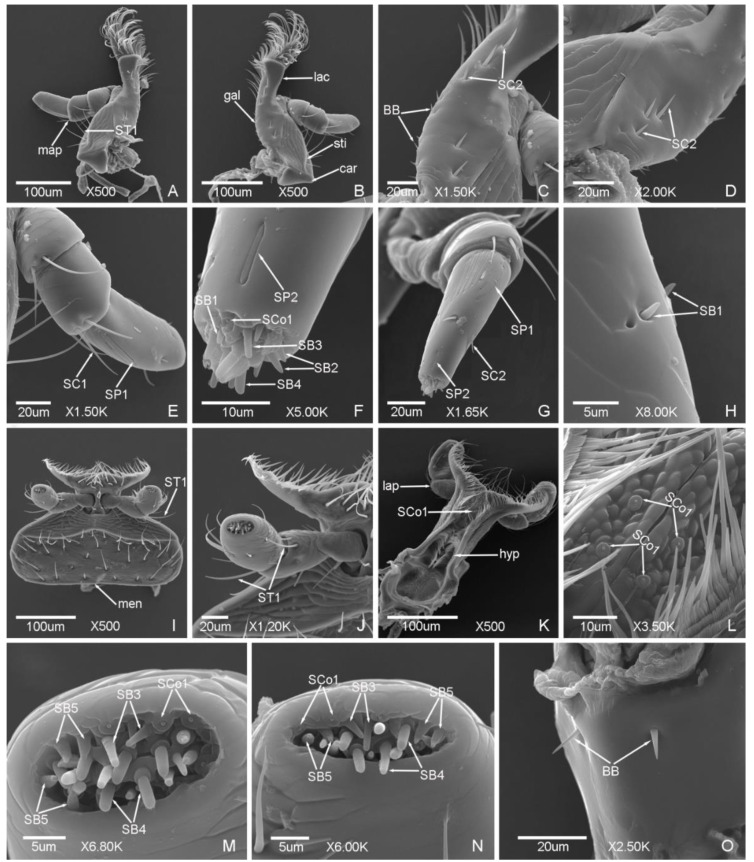
Sensilla on the maxillae and labium of *Meligethes* (*Odonthogethes*) *chinensis* female. (**A**,**B**) Ventral view of maxillae. map, maxillary palps; car, cardo; sti, stipes; gal, galea; lac, lacinia; ST1, sensilla trichodea 1; (**C**,**D**) the sensilla on the galea. SC2, sensilla chaetica 2; BB, Böhm bristles; (**E**–**H**) the sensilla on the maxillary palps. SC1 and SC2, sensilla chaetica 1, 2; SP1 and SP2, sensilla placodea 1 and 2; SB1, SB2, SB3 and SB4, sensilla basiconica 1, 2, 3 and 4; SCo1, sensilla coeloconica 1; (**I**,**J**) the ventral view of labium and mentum. men, mentum; ST1; (**K**,**L**) the dorsal view of labium. lap, labial palps; hyp, hypopharynx; SCo1; (**M**,**N**) the sensilla on the tip of the terminal segment of labial palps. SB3, SB4 and SB5, sensilla basiconica 3, 4 and 5; SCo1; (**O**) BB on the labium.

*Meligethes* (*Odonthogethes*) *chinensis* has typical biting mouthparts composed of labrum, mandible, maxillae, labium, and hypopharynx. Sensilla are mainly presented on the labrum, maxillary palps, and labial palps, while they are less presented on the mandible (Figure 6, Figure 7, Figure 8 and Figure 9).

Seven types of sensilla are observed on the mouthparts of the male (Figure 6 and Figure 7) and female adults (Figure 8 and Figure 9) which are composed of seventeen subtypes, including sensilla trichodea (two subtypes), sensilla chaetica (two subtypes), sensilla basiconica (seven subtypes), sensilla coeloconica (two subtypes), sensilla placodea (two subtypes), Böhm bristles, and sensilla campaniformia (Table 3; Figure 6, Figure 7, Figure 8 and Figure 9). There is a small difference in the sensilla on the mouthparts of males and females. One subtype of sensilla basiconica (SB6) was found in males only, and SCo2 present sex dimorphism, while other types of sensilla are similar in males and females.

#### 3.2.1. Sensilla Trichodea (ST)

ST are long and thin, with a slightly grooved surface, thin tip, and shallow socket. ST were found in both males and females. They can be divided into two subtypes according to the length.

ST1 are long, with a sharp and curved tip, and presented on the lateral side of stipes (Figure 7a and Figure 9a) and labial palps (Figure 7i,k and Figure 9i,j). The length was 33.81 ± 3.91 μm and the base diameter was 2.25 ± 0.14 μm.

ST2 are shorter than ST1, with a slightly curved tip, and presented on the lateral side of the mandible only (Figure 6c and Figure 8d). The length was 22.38 ± 3.00 μm and the base diameter was 1.72 ± 0.09 μm.

#### 3.2.2. Sensilla Chaetica (SC)

SC are straight, with a slightly grooved surface and shallow socket. The tip is sharper than sensilla trichodea. SC are presented on the maxillae of both males and females, and can be divided into two subtypes according to the length.

SC1 are long and bristly, and presented on the maxillary palps (Figure 7c and Figure 9e). The length was 31.39 ± 1.79 μm and the base diameter was 2.35 ± 0.17 μm.

SC2 are shorter than SC1, with a tapering tip, and presented on the maxillary palps and galea. The length and base diameter of SC2 on maxillary palps were 11.56 ± 0.02 μm and 2.57 ± 0.06 μm, respectively (Figure 7c and Figure 9g), while the length and base diameter of SC2 on galea were 8.70 ± 0.05 μm and 1.73 ± 0.04 μm, respectively (Figure 7d and Figure 9c,d).

#### 3.2.3. Sensilla Basiconica (SB)

SB are thick and straight, with a cone shape, and the surface is smooth or patterned near the tip (SB5). The base is mostly cone-shaped or mound like, or with a deep socket (SB7). SB are mainly presented on the maxillary palps, labial palps, and epipharynx. Seven subtypes were found in this study. Except for SB6, which were found in males only, other subtypes were found in both males and females.

SB1 are short and small, with a blunt tip and deep socket, and presented on the tip of the maxillary palps (Figure 7f and Figure 9f) and on the side of the terminal segment of the maxillary palps (Figure 9h). The length was 2.54 ± 0.10 μm and the base diameter was 1.06 ± 0.09 μm.

SB2 are longer than SB1, with a sharp tip and shallow socket, and presented on the tip of the maxillary palps (Figure 7e,f and Figure 9f). The length was 3.87 ± 0.55 μm and the base diameter was 1.48 ± 0.14 μm.

SB3 with a round tip, and shallow socket. SB3 are located on the mound-like base, and presented on the tip of the maxillary palps (Figure 7e,f and Figure 9f) and the tip of the labial palps (Figure 7l,m and Figure 9m,n). The length was 4.50 ± 0.98 μm and the base diameter was 1.24 ± 0.18 μm.

SB4 are short and thick, thicker than SB3, with a blunt tip and shallow socket, and located on the mound-like base. SB4 presented on the tip of the maxillary palps (Figure 7g and Figure 9f) and the tip of the labial palps (Figure 7l,m and Figure 9m,n). The length was 4.85 ± 0.21 μm and the base diameter was 1.94 ± 0.17 μm.

SB5 with a small round protuberance and patterned surface. The distal of SB5 is slightly curved. SB5 are located on the mound-like protuberance base and shallow socket, and presented on the tip of the labial palps (Figure 7l,m and Figure 9m,n). The length was 3.76 ± 0.15 μm and the base diameter was 1.64 ± 0.05 μm.

SB6 are thick and long, with a shallow socket and a groove on the tip. SB6 are located on the cone-shaped protuberance base, and presented on the tip of the maxillary palps (Figure 7e,f) and the tip of the labial palps (Figure 7m) of the males. The length was 3.76 ± 0.15 μm and the base diameter was 1.64 ± 0.05 μm.

SB7 are short, conical, with a sharp tip and deep socket, and symmetrically presented on the epipharynx (Figure 6e,f and Figure 8g). The length was 1.91 ± 0.11 μm and the base diameter was 1.32 ± 0.07 μm.

#### 3.2.4. Sensilla Coeloconica (SCo)

SCo are cone-shaped, with a blunt tip, and located on a deep and obvious socket. SCo are presented on the same position of males and females, and can be divided into two subtypes according to the size of cone and the width of socket.

SCo1 are short and small, with a round protuberance and smooth surface, and presented on the tip of the terminal segments of maxillary palps (Figure 7e,f and Figure 9f) and labial palps (Figure 7l,m and Figure 9m,n). SCo1 are also symmetrically presented on the middle of the dorsal view of the labium and the quantity was four (Figure 7h,j and Figure 9k,l). The length was 0.60 ± 0.07 μm and the base diameter was 2.19 ± 0.15 μm.

SCo2 are significantly longer than SCo1, with a deep grooved surface and blunt flat tip, and presented on both sides of the wall of the ventral view of labrum (Figure 6g), five of SCo2 on each side (Figure 6h and Figure 8h). There is sex dimorphism, SCo2 in males and females are obviously different, one side of the cone is close to the wall of the socket in the males (Figure 6h), but not close in the females (Figure 8h). The length was 3.91 ± 0.27 μm and the base diameter was 2.64 ± 0.20 μm.

#### 3.2.5. Sensilla Placodea (SP)

SP are straight and embedded in the sunken epidermis, with a smooth surface and shallow socket, and mainly presented on the terminal segments of the maxillary palps of both males and females. According to the quantity and the distribution position, SP can be divided into two subtypes.

SP1 are long, with the quantity of seven on each outer side of the terminal segments of maxillary palps (Figure 6a and Figure 9e,g). The length was 21.88 ± 2.11 μm and the base diameter was 0.87 ± 0.14 μm.

SP2 are shorter than SP1, only one presented on the distal of the terminal segments of maxillary palps (Figure 7g and Figure 9f,g). The length was 8.64 ± 0.16 μm and the base diameter was 1.19 ± 0.13 μm.

#### 3.2.6. Böhm Bristles (BB)

BB are short and straight, no hole, with a smooth surface, sharp tip, and deep socket. BB are mainly presented on the lateral side of the galea of both males and females (Figure 7d and Figure 9c). The length was 5.39 ± 0.67 μm and the base diameter was 1.26 ± 0.08 μm. BB also presented on the middle of the ventral view of the labium (Figure 7n and Figure 9o). The length was 10.59 ± 0.99 μm and the base diameter was 1.74 ± 0.18 μm.

#### 3.2.7. Sensilla Campaniformia (SCam)

SCam are bell-shaped, semi-elliptical or hemispherical, with a smooth surface, thick wall edge and deep socket, and located on a small round cavity. One SCam on each side, almost symmetrically presented on the base of the ventral view of labrum of both males and females (Figure 6g,i and Figure 8f). The length and base diameter of SCam were 1.58 ± 0.04 μm and 1.81 ± 0.09 μm, respectively. The diameter of the socket was 3.33 ± 0.18 μm.

## 4. Discussion

### 4.1. Comparison of the Sensilla on the Antennae and Mouthparts between Saprophagous and Phytophagous Nitidulidae

In this study, we reported the sensilla on the antennae and mouthparts of both males and females of the pollen-beetle *Meligethes* (*Odonthogethes*) *chinensis*. This is the first focus on the sensilla of the phytophagous (anthophagous) Nitidulidae. Comparing with the sensilla of the saprophagous Nitidulidae (*Omosita colon*) reported by Cao and Huang [14], there are obvious differences on the antennae and mouthparts (Table 4 and Table 5).

Cao and Huang [14] found six types of sensilla on the antennae of both males and females of *O. colon*, including sensilla trichodea (ST; three subtypes), sensilla chaetica (SC; two subtypes), sensilla basiconica (SB; three subtypes), Böhm bristles (BB), sensilla styloconica (SS), and sensilla cavity (SCa). In this study, in addition to the above six types of sensilla observed on the antennae of both males and females of *M. chinensis*, another type of sensilla (SCo) was also found on the antennae of females of *M. chinensis*, which mainly presented on the antennal terminal segment. There were also significant differences in the sensilla subtypes. In this study, we found the leaf-like sensilla chaetica (SC2) on the scape of females; the short and thick sensilla styloconica (SS3) on the antennal terminal segment of both males and females, and the nailed sensilla styloconica with bifurcated tip (SS5) in the males; the sensilla styloconica with small protuberance (SS2), and the nailed sensilla styloconica with sharp tip (SS4) in the females of *M. chinensis*. Although the number of these newly described sensilla subtypes in Nitidulidae was very small, the position of the same kind of sensilla subtypes was very concentrated.

Cao and Huang [14] found seven types of sensilla on the mouthparts of *Omosita colon*, including sensilla trichodea, sensilla chaetica (two subtypes), sensilla basiconica (seven subtypes), sensilla coeloconica, sensilla placodea (two subtypes), Böhm bristles, and sensilla campaniformia (two subtypes). In this study, we found that the seven types of sensilla on the mouthparts of *M. chinensis* were the same as those of *O. colon*. Among them, sensilla placodea and Böhm bristles had almost no difference in morphological characteristics in *M. chinensis* and *O. colon*, and sensilla chaetica with a sharp tip in *M. chinensis* were the same as SC1 in *O. colon*. However, sensilla trichodea had a slightly grooved surface in *M. chinensis* but a toothed surface in *O. colon*. Sensilla campaniformia found in *M. chinensis* were longer, and the socket was deeper than *O. colon*. The subtypes of sensilla basiconica and sensilla coeloconica were significantly different in *M. chinensis* and *O. colon*. For instance, the sensilla basiconica with a groove at the top (SB6) were found in the males of *M. chinensis* only, and the sensilla coeloconica with deep grooved surface (SCo2) were found in both males and females of *M. chinensis*. SB6 and SCo2 were not found in *O. colon*. In addition, the arrangement of SB7 was obviously different on the ventral labrum in *M. chinensis* and *O. colon* (Figures 6e and 7b in Cao and Huang, 2016) [14].

### 4.2. Comparison of the Sensilla on the Antennae and Mouthparts in Coleoptera

In this study, nine types of the sensilla (ST, SC, SB, BB, SS, SCa, SCo, SP, SCam) were found on the antennae and mouthparts in *M. chinensis*. Their identification and naming mainly refer to Schneider 1964, Cao and Huang 2016 [14,17], and to additional articles published on the following families or the superfamily of Coleoptera: Cerambycidae [19,20], Coccinellidae [12,21], Chrysomelidae [22,23], Curculionidae [24,25], Carabidae [26,27], Elateridae [28,29] and Scarabaeoidea [30,31].

For some sensilla present only in a small number, their characteristics were unstable, so their naming was difficult to be consistent in different taxa. For instance, the grooved and nail-shaped sensilla (SS4 and SS5) found in *M. chinensis* were obviously different from the grooved “GP” in *Callosobruchus maculatus* (Chrysomelidae, Bruchinae, Figure 3f in Hu et al., 2009) [32], so they were classified as the subtypes of SS in this study. The bell-shaped sensilla (SCam) presented on the ventral labrum in *M. chinensis* were similar with the “SCa” on the antennae of *Holotrichia parallela* (Scarabaeidae, Figure 1i in Du et al., 2015) [33], and the “sensilla coeloconica” on the antennae of *Oryctes nasicornis* (Scarabaeidae, Figure 3m in Bohacz et al., 2020) [31]. We named them as SCam in this study in order to keep the naming of sensilla in the same family consistent, although they are longer and their sockets are deeper than the “SCam1” in *O. colon* (Nitidulidae, Figure 7d in Cao and Huang, 2016) [14]. The short, cone-shaped sensilla (SC2) presented on the antennal scape in *M. chinensis* were similar with the “leaf-shaped sensilla” on the antennae of *Liparetus obscurus* (Scarabaeidae, Figure 3w in Bohacz et al., 2020) [31]. However, considering that only two of this kind of short cone-shaped sensilla were found in *M. chinensis*, and they were very similar to SC1 in Nitidulidae (only the length is different), so they were regarded as a subtype of SC (SC2) in this study.

### 4.3. Inference of Sensilla Function on the Antennae and Mouthparts of Meligethes (Odonthogethes) chinensis

As reported above, nine types of the sensilla (ST, SC, SB, BB, SS, SCa, SCo, SP, SCam) were found on the antennae and mouthparts in *M. chinensis*. ST are usually considered as tactile sensilla [19]. Bartlet et al. (1999) [22] found that ST in *Psylliodes chrysocephala* (Chrysomelidae, Alticinae) also have an olfactory function and play an important role in the sense of smell of the host [34]. ST are presented in each segment of the antennae of *M. chinensis*, which may be used to perceive olfactory cues of the host plant in addition to tactile signals. SC are generally considered as the mechanical sensilla receiving external stimuli (Schneider, 1964) [17]. SC1 are significantly longer than other types of sensilla on the lateral side of the antennal club and maxillary palps of males and females, and there are short and thin SC2 on the maxillary palps and galea of both sexes of *M. chinensis*, probably in order to get in contact with external stimuli faster. However, the short, leaf-shaped SC2 are presented on the lateral side of the scape of females only and may help control the curvature of scape and sense movement [35]. SB are usually considered as olfactory receptors. In *M. chinensis*, SB can be divided into three subtypes on the antennae and seven subtypes on the mouthparts. Except SB6, which presented on the tip of the maxillary palps and the tip of the labial palps of the males only, other subtypes are found in both males and females. SB1 and SB2 on the antennae are, respectively, similar with the “SB1” and “SB2” on the antennae of *Callosobruchus maculatus* (Chrysomelidae, Bruchinae, Figure 3d,e in Hu et al., 2009) [32]. These subtypes of sensilla may have olfactory function (Zacharuk, 1985) [36]. The slightly grooved SB3 are thicker than SB1 on the antennae, we inferred that they may be the specialization of SB1, also having olfactory function. It is inferred that SB1, SB2, and SB3 on the mouthparts are probably used as gustatory or olfactory receptors (Cao and Huang, 2016) [14]. SB4 and SB5 are, respectively, similar with the “SB2” and “SB1” in *Siagona europaea* (Carabidae, Figure 1d,e in Giglio et al., 2010) [27], and they may be olfactory receptors. The newly found subtype SB6 in Nitidulidae are similar to the “SB8” with a terminal pore on the antennae of *Selasia* sp. (Elateridae, Figure 9b in Faucheux and Kundrata, 2017) [29], and they may be taste receptors in males of *M. chinensis* that respond to air stimulation. SB7 are similar with the “SB5” in *Carabus prodigus* (Carabidae, Figure 1o in Liu and Tian, 2008) [37]; they may be other olfactory receptors. BB are gravity receptors to buffer force when insects are stimulated by mechanical forces. In *M. chinensis*, BB are usually located at the base of scape and pedicel, and on the lateral side of the galea, also presented on the middle of the ventral surface of the labium of both males and females, in order to sense the falling speed of antennae and mouthparts position [17,38]. SCa are sensilla generally considered to have an important role as humidity and temperature detectors (Stange and Stowe, 1999) [39]. Davis and Sokolove (1975) [40] found that there are temperature and humidity receptor cells in SCo. Giglio et al. (2010) [27] confirmed that SCo are temperature and humidity sensilla. SCo on the antennae were found in the females of *M. chinensis* only, which may help females to perceive the temperature and humidity of the habitat to find a suitable oviposition site on the host plant. On the other hand, SCo2 on the labrum present sexual dimorphism. SCo2 on females extend more outwardly than males, which may help females to be sensitive to temperature and humidity, thereby providing possibilities for them to select food and oviposition sites. SS are generally considered as sex chemical receptors, probably with an additional function of temperature or humidity receptors [22,41]. There are different subtypes of SS on the antennae of males and females in *M. chinensis*, which may help them identify the sex chemicals released by the opposite sex effectively. SP were speculated to be olfactory receptors, and related to searching for host habitats [42,43]. SP presented on the terminal segments of maxillary palps in *M. chinensis*, making it easy for them to find and identify the host plant. In *M. chinensis*, SCam located in the round cavity may be a kind of temperature and humidity sensilla, and they are also considered as mechanoreceptors, which are not innervated and can respond to the deformation of the epidermis [41,44,45].

## 5. Conclusions

There are many types of sensilla in Coleoptera, but the nomenclature is not uniform, which brings some difficulties to the identification of sensilla. This study analyzes the sensilla on the antennae and mouthparts of the pollen-beetle *Meligethes (Odonthogethes) chinensis* (an anthophagous insect among Nitidulidae) for the first time. More research should be carried out to explore the different types of sensilla present in this family, in order to standardize the nomenclature and subtype division of sensilla on antennae and mouthparts of Nitidulidae. Further analyses are also necessary to better clarify the functions of several types of sensilla, thus providing evidence also on the evolution of the highly heterogeneous feeding habits in Nitidulidae.

## Figures and Tables

**Table 1:** Length of antennal segments of *Meligethes* (*Odonthogethes*) *chinensis* males and females.

Sex	Scape (μm)	Pedicel (μm)	Flagellomeres (μm)									Total (μm)
			F1	F2	F3	F4	F5	F6	F7	F8	F9	
male	100.09 ± 3.92	72.94 ± 1.72	63.84 ± 2.73	41.17 ± 2.04	38.21 ± 2.15	28.36 ± 2.03	26.54 ± 0.80	25.55 ± 1.09	42.53 ± 0.24	38.48 ± 0.12	87.68 ± 0.46	565.37 ± 3.76
female	105.34 ± 5.34	69.42 ± 0.36	60.98 ± 1.73	42.66 ± 0.37	42.19 ± 3.09	32.52 ± 0.89	27.24 ± 1.71	27.11 ± 1.09	47.29 ± 0.20	42.96 ± 0.80	95.25 ± 2.17	593.96 ± 9.65

The length of antennal segments is the mean length (mean ± SE) from eight male and eight female adults.

**Table 2:** Morphological types of antennal sensilla on *Meligethes* (*Odonthogethes*) *chinensis*.

Types of Sensilla	Morphological Types of Sensilla					
	Length (μm)	Diameter (μm)	Tip	Wall	Socket	Shape
ST1	24.35 ± 3.30	1.30 ± 0.12	Sharp	Grooved	Tight	Slightly curved
ST2	13.41 ± 1.80	1.67 ± 0.11	Sharp	Grooved	Tight	Curved
ST3	14.91 ± 1.09	2.12 ± 0.13	Blunt	Smooth	Tight	Straight
SC1	25.82 ± 0.83	2.50 ± 0.10	Sharp	Grooved	Tight	Straight
SC2	8.28 ± 0.08	1.96 ± 0.10	Slightly sharp	Smooth	Tight	Straight and leaf-like
SB1	7.66 ± 0.65	2.94 ± 0.17	Blunt	Smooth	Tight	Straight or slightly curved
SB2	5.62 ± 0.38	2.93 ± 0.11	Blunt	Grooved	Tight	Straight
SB3	7.03 ± 0.53	2.99 ± 0.23	Blunt	Grooved	Tight	Straight
BB	4.84 ± 0.65	1.47 ± 0.11	Sharp	Smooth	Wide	Straight
SS1	4.22 ± 0.21	2.45 ± 0.03	Sharp	Smooth	Tight	Straight
SS2	2.64 ± 0.11	2.56 ± 0.09	Blunt	Smooth	Tight	Straight
SS3	1.26 ± 0.16	3.38 ± 0.28	Blunt	Smooth	Tight	Straight
SS4	4.18 ± 0.05	3.03 ± 0.16	Sharp	Smooth	Tight	Straight and nail like
SS5	3.10 ± 0.05	2.92 ± 0.10	Bifurcated	Smooth	Tight	Straight and nail like
SCa	\	2.03 ± 0.12	\	\	\	Concave cavity
SCo	1.53 ± 0.03	2.81 ± 0.15	Blunt	Smooth	Wide	Straight

The length and diameter are the mean length and diameter (mean ± SE) of at least eight sensilla for each type.

**Table 3:** Morphological types of sensilla on the mouthparts of *Meligethes* (*Odonthogethes*) *chinensis*.

Types of Sensilla	Morphological Types of Sensilla					
	Length (μm)	Diameter (μm)	Tip	Wall	Socket	Shape
ST1	33.81 ± 3.91	2.25 ± 0.14	Sharp	Grooved	Tight	Slightly curved
ST2	22.38 ± 3.00	1.72 ± 0.09	Sharp	Grooved	Tight	Slightly curved
SC1	31.39 ± 1.79	2.35 ± 0.17	Sharp	Grooved	Tight	Straight
SC2 (maxillary palps)	11.56 ± 0.02	2.57 ± 0.06	Sharp	Smooth	Tight	Straight
SC2 (galea)	8.70 ± 0.05	1.73 ± 0.04	Sharp	Smooth	Tight	Straight
SB1	2.54 ± 0.10	1.06 ± 0.09	Blunt	Smooth	Wide	Straight
SB2	3.87 ± 0.55	1.48 ± 0.14	Blunt	Smooth	Tight	Straight
SB3	4.50 ± 0.98	1.24 ± 0.18	Blunt	Smooth	Tight	Straight
SB4	4.85 ± 0.21	1.94 ± 0.17	Blunt	Smooth	Tight	Straight
SB5	3.76 ± 0.15	1.64 ± 0.05	Blunt	Patterned	Tight	Slightly curved
SB6	4.68 ± 0.17	1.81 ± 0.02	Grooved	Smooth	Tight	Straight
SB7	1.91 ± 0.11	1.32 ± 0.07	Sharp	Smooth	Wide	Straight
SCo1	0.60 ± 0.07	2.19 ± 0.15	Blunt	Smooth	Wide	Straight
SCo2	3.91 ± 0.27	2.64 ± 0.20	Blunt	Grooved	Wide	Curved
SP1	21.88 ± 2.11	0.87 ± 0.14	Blunt	Smooth	Tight	Straight
SP2	8.64 ± 0.16	1.19 ± 0.13	Blunt	Smooth	Tight	Straight
BB (galea)	5.39 ± 0.67	1.26 ± 0.08	Sharp	Smooth	Wide	Straight
BB (labium)	10.59 ± 0.99	1.74 ± 0.18	Sharp	Smooth	Wide	Straight
SCam	1.58 ± 0.04	1.81 ± 0.09	Blunt	Smooth	Wide	Semi-elliptical or hemispherical

The length and diameter are the mean length and diameter (mean ± SE) of at least eight sensilla for each type.

**Table 4:** Comparison of the antennal sensilla of *Omosita colon* and *Meligethes* (*Odonthogethes*) *chinensis*.

Species	Feed Habit	Sex	ST	SC	SB	BB	SS	SCa	SCo	Total
*Omosita colon* (in Cao and Huang (2016))	Saprophagy (corpses of bovine)	Male Female	3	2	3	1	1	1	\	6 types 11 subtypes
*Meligethes* (*Odonthogethes*) *chinensis* (in this study)	Phytophagy (Host plant: *Rubus idaeus* flowers)	Male	3	1	3	1	3	1	\	6 types 12 subtypes
		Female	3	2	3	1	3	1	1	7 types 14 subtypes

**Table 5:** Comparison of the sensilla on the mouthparts of *Omosita colon* and *Meligethes* (*Odonthogethes*) *chinensis*.

Species	Feed Habit	Sex	ST	SC	SB	SCo	SP	BB	SCam	Total
*Omosita colon* (in Cao and Huang (2016))	Saprophagy (corpses of bovine)	Male	1	2	7	1	2	1	2	7 types 16 subtypes
*Meligethes* (*Odonthogethes*) *chinensis* (in this study)	Phytophagy (Host plant: *Rubus idaeus* flowers)	Male	2	2	7	2	2	1	1	7 types 17 subtypes
		Female	2	2	6	2	2	1	1	7 types 16 subtypes
